# Biofuels: From Microbes to Molecules (Book Review)

**DOI:** 10.3389/fbioe.2015.00016

**Published:** 2015-02-16

**Authors:** M. Kalim Akhtar, Patrik R. Jones

**Affiliations:** ^1^Institute of Molecular Plant Sciences, School of Biological Sciences, University of Edinburgh, Edinburgh, UK; ^2^Department of Life Sciences, Imperial College London, London, UK

**Keywords:** biofuel, synthetic biology, metabolic engineering, hydrogen, alcohol, fatty acid, alkane, cyanobacteria

A steady and continuous supply of energy will be pivotal if we are to sustain the infrastructure necessary for the current and future development of modern societies. Almost 80% of our current energy supply is derived from the combustion of fossil fuels and, even taking into account the discovery of new reserves, it is predicted that fossil fuel supplies will be severely depleted over the next century concomitant with an increase in energy demand (Sawin and Sverrisson, 2014). Furthermore, the observed rise in global temperatures, which has been consensually attributed in part to increased fossil fuel combustion, may well have adverse long-term climatic and environmental impact. Is there a solution to any of this?

Well, microbes could possibly hold the answer. A book recently entitled, “Biofuels: From Microbes to Molecules” edited by Lu (2014) describes the synthesis of biofuels using microbes. Future technologies, based upon microbial engineering, could in principle lead to the development of sustainable and environment-friendly approaches for the production of fuels. Centered on this benign approach emerge a number of key questions. What fuels could be manufactured in this way? How is it possible for biologists to engineer microbes to generate fuels? How efficient could we make this process? Would it be sustainable enough to meet our global energy demands? This book addresses these all-important questions.

*Biofuels: From Microbes to Molecules* is a timely compilation of highly focused reviews written by leading experts in the field of microbial biofuel technology. It is aimed for the advanced student or researchers with a firm grounding in the basic concepts and principles of biology. Each chapter covers the general background, research challenges, and future prospect of biofuel production, as well as its technical aspects including the design and construction of biosynthetic pathways, optimization of genetic and regulatory processes, enzyme engineering, and the employment of various hosts such as bacteria, yeast, and microalgae. Seven of the chapters focus on a specific biofuel candidate and these include hydrogen, biogas, ethanol, butanol, higher chain alcohols, isoprene, and fatty-acid derived biofuels. Since photosynthetic organisms are expected to form the cornerstone of any microbe-based fuel economy, the final chapter discusses recent advances on the use of cyanobacteria as hosts for the production of fatty-acid derived products.

Regarding the editor, Xuefeng Lu is currently serving as the Deputy Director General of *Qingdao Institute of Bioenergy and Bioprocess Technology*. He has done a commendable job on assembling a top-notch panel of international experts, undertaking research in various parts of the world including Canada, China, Germany, Hungary, Korea, and South Africa. The field of bioenergy is undoubtedly a fast-growing and hugely expanding research area that has led to a wealth of information particularly in recent years. As researchers, we are all too aware that trawling through the literature can be a time-consuming and laborious affair. By compiling and summarizing our current knowledge on microbial biofuels, the authors have provided us with a rich literary material of excellent depth, coverage, and clarity. In addition to being a highly recommended read for experts and students in the field, this book further serves as a testament to the phenomenal progress that has been made in microbial engineering over the last decade.

## Conflict of Interest Statement

The authors declare that the research was conducted in the absence of any commercial or financial relationships that could be construed as a potential conflict of interest.
